# Genomic Analysis of *Mycobacterium tuberculosis* Strains Resistant to Second-Line Anti-Tuberculosis Drugs in Lusaka, Zambia

**DOI:** 10.3390/antibiotics12071126

**Published:** 2023-06-29

**Authors:** Joseph Yamweka Chizimu, Eddie Samuneti Solo, Precious Bwalya, Thoko Flav Kapalamula, Kaemba Kunkuta Mwale, David Squarre, Misheck Shawa, Patrick Lungu, David Atomanyi Barnes, Kaunda Yamba, Tiza Mufune, Herman Chambaro, Harvey Kamboyi, Musso Munyeme, Bernard Mudenda Hang’ombe, Nathan Kapata, Victor Mukonka, Roma Chilengi, Jeewan Thapa, Chie Nakajima, Yasuhiko Suzuki

**Affiliations:** 1 Division of Bioresources, Hokkaido University International Institute for Zoonosis Control, Sapporo 001-0020, Hokkaido, Japan; chizimuyjoseph@yahoo.com (J.Y.C.); kamboyihk@czc.hokudai.ac.jp (H.K.);; 2 Zambia National Public Health Institute, Ministry of Health, Lusaka 10101, Zambia; nkapata@gmail.com (N.K.); chilengir@yahoo.com (R.C.); 3 University Teaching Hospital, Ministry of Health, Lusaka 10101, Zambia; samuneti2@yahoo.com (E.S.S.); kaundayamba@gmail.com (K.Y.); 4 Department of Pathobiology, Faculty of Veterinary Medicine, Lilongwe University of Agriculture and Natural Resources, Lilongwe 207203, Malawi; 5 Department of Veterinary Services, Ministry of Fisheries and Livestock, Lusaka 10101, Zambia; 6 National TB Control Program, Ministry of Health, Lusaka 10101, Zambia; 7 Provincial Health Office, Central Province, Ministry of Health, Kabwe 10101, Zambia; 8 Department of Disease Control, School of Veterinary Medicine, The University of Zambia, Lusaka 10101, Zambia; 9 Department of Para-Clinical Studies, School of Veterinary Medicine, The University of Zambia, Lusaka 10101, Zambia; bhangombe@unza.zm; 10 School of Public Health and Environmental Sciences, Levy Mwanawasa Medical University, Ministry of Health, Lusaka 10101, Zambia; vmukonka@gmail.com; 11 International Collaboration Unit, Hokkaido University International Institute for Zoonosis Control, Sapporo 001-0020, Hokkaido, Japan; 12 Institute for Vaccine Research and Development, Hokkaido University, Sapporo 001-0020, Hokkaido, Japan

**Keywords:** *Mycobacterium tuberculosis*, pre-extensively drug resistance, recent transmission, whole-genome sequencing, Zambia

## Abstract

The emergence of pre-extensively drug-resistant tuberculosis (pre-XDR-TB) is a threat to TB control programs in developing countries such as Zambia. Studies in Zambia have applied molecular techniques to understand drug-resistance-associated mutations, circulating lineages and transmission patterns of multi-drug-resistant (MDR) *Mycobacterium tuberculosis*. However, none has reported genotypes and mutations associated with pre-XDR TB. This study characterized 63 drug-resistant *M. tuberculosis* strains from the University Teaching Hospital between 2018 and 2019 using targeted gene sequencing and conveniently selected 50 strains for whole genome sequencing. Sixty strains had resistance mutations associated to MDR, one polyresistant, and two rifampicin resistant. Among MDR strains, seven percent (4/60) had mutations associated with pre-XDR-TB. While four, one and nine strains had mutations associated with ethionamide, para-amino-salicylic acid and streptomycin resistances, respectively. All 50 strains belonged to lineage 4 with the predominant sub-lineage 4.3.4.2.1 (38%). Three of four pre-XDR strains belonged to sub-lineage 4.3.4.2.1. Sub-lineage 4.3.4.2.1 strains were less clustered when compared to sub-lineages L4.9.1 and L4.3.4.1 based on single nucleotide polymorphism differences. The finding that resistances to second-line drugs have emerged among MDR-TB is a threat to TB control. Hence, the study recommends a strengthened routine drug susceptibility testing for second-line TB drugs to stop the progression of pre-XDR to XDR-TB and improve patient treatment outcomes.

## 1. Introduction

Globally 4000 lives are lost in a day due to tuberculosis (TB) making it one of the major infectious causes of mortality [1] and a public health threat. In spite of the global downward trend in annual TB notifications which was attributed to the COVID-19 pandemic, Zambia experienced an upward trend [1]. In 2021, the country recorded a TB incidence of 59,000, with 484 laboratories confirming multi-drug-resistant or rifampicin-resistant TB (MDR/RR-TB). Additionally, Zambia had more than a double increment in the estimated percentage of TB cases with MDR/RR-TB among the new cases from 2015 to 2017 [2,3,4] The country was classified by WHO as one of the countries with a high TB burden, HIV/TB coinfection and MDR/RR-TB [1]. MDR-TB is defined as TB resistant to both isoniazid and rifampicin, while pre-extensively drug-resistant TB (pre-XDR), as TB resistant to any fluoroquinolone (a class of second-line anti-TB drug) and rifampicin. Whereas extensively drug-resistant (XDR) TB refers to TB resistant to any fluoroquinolone and rifampicin plus at least one of the drugs bedaquiline and linezolide [1].

Drug resistance develops as a result of *M. tuberculosis* spontaneous gene mutations which cause the bacteria to become resistant to the most widely used anti-TB drugs [5]. This is due to a number of reasons including prescription of ineffective treatment regimens, poor compliance or the failure to ensure that patients finish the entire course of treatment [6,7]. Most common point mutations associated with resistance to second-line drugs [8] such as fluoroquinolones occur in the conserved quinolone resistance-determining region (QRDR) of *gyrA* and *gyrB* genes [9]. While mutations in the *atpE* and *rrs* genes are associated with resistance to bedaquiline and aminoglycosides such as kanamycin, respectively [5].

According to the current tuberculosis treatment guidelines in Zambia [10], all drug-resistant patients should have resistant profiles to second-line TB drugs (levofloxacin, moxifloxacin, clofazimine, bedaquiline and linezolid) carried out to guide patient management. However, this has been a challenge due to inadequate diagnostic capacity in the country despite the increasing number of patients resistant to these drugs. As such, some patients are subjected to inappropriate treatment regimens leading to the selection and evolution of drug-resistant strains [11,12], such as the progression of pre-XDR to XDR.

The two main causes of tuberculosis in humans are broadly known as M. tuberculosis and M. africanum [13]. These have seven major lineages which are able to maintain full infection, disease, and transmission cycles in humans [14]. They differ from each other as a result of varied compositions of single nucleotide polymorphisms (SNPs), insertion and deletion (indels), large genomic deletions, and duplications [15]. The lineages are Lineages one to four and seven (L1 to L4, L7) under M. tuberculosis, and five to six (L5-L6) under M. africanum [13]. These lineages have been found to have distinct geographical dispensations, with L2 and L4 having a global distribution, L1 around the Indian Ocean, and L3 in East Africa, Central and South Asia. In contrast, L5 and L6 are confined to certain geographical regions, mainly West Africa. The increased fitness of the pathogen in a certain host population it has co-evolved with has been attributed to these lineages’ geographic preferences [16]. It is crucial to have knowledge about the circulating lineages in a given area because they differ in terms of immunogenicity, pathogenicity, transmissibility, clinical outcomes of tuberculosis and the development of drug resistance [15,17].

In Zambia, previous studies revealed the major strains sub-lineage 3.1.1 (SIT21/CAS1-Kili) and sub-lineage 4.3.4.1 (SIT20/LAM1) to be responsible for MDR-TB, and recent transmission among the strains was collected from 2013 to 2017 [18,19]. While sublineage 4.3.4.2.1 (SIT59/LAM11_ZWE) was the most prevalent sub-lineage [18,19] similar to the report by Mulenga et al., 2010 [20], indicating its predominance for over a decade. Fortunately, the association of the predominant sub-lineage 4.3.4.2.1 with MDR-TB was not significant [18] and, none of the studies in Lusaka reported the presence of pre-XDR-TB *M. tuberculosis* strains and its genotypes.

In this study, we report the emerging of second-line drug-resistant *M. tuberculosis* genotypes in Lusaka, a densely populated city in Zambia, using whole genome sequencing.

## 2. Results

### 2.1. Study Samples

**Figure 1:**
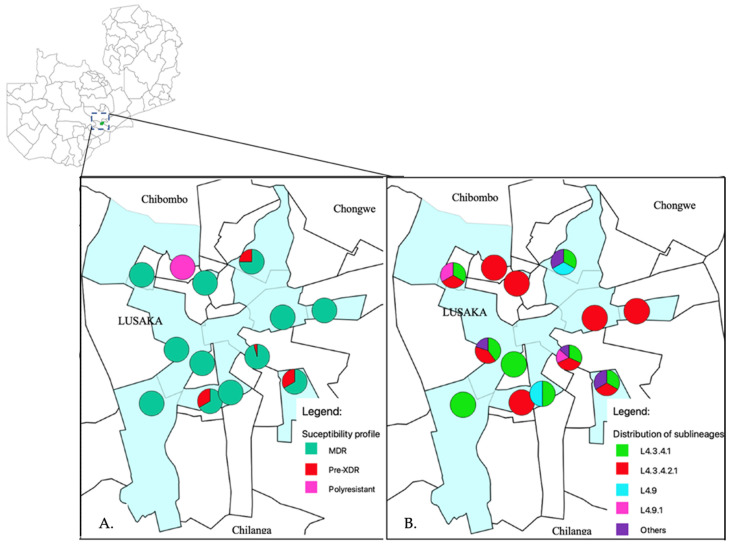
The administrative wards where the health facilities are located that referred patients to the University Teaching Hospital, are highlighted in light blue. (**A**) Shows distribution of MDR and pre-XDR strains in Lusaka. In the pie charts, green, red and pink represents MDR, pre-XDR and polyresistant phenotypes, respectively. The pie charts were placed on a map using the global position system (GPS) coordinates for the health facilities. (**B**) The distribution of *M. tuberculosis* sub-lineages in Lusaka district. In the pie charts the colors; Red, green, light blue, pink and purple represents sub-lineages L4.3.4.1, L4.3.4.2.1, L4.9, L4.9.1 and others, respectively.

Of the studied 63 samples, 41 were from male and 15 from female patients aged between 20 to 78 years old. Many of these 56% (35/63) were below 40 years old. The majority 78% (49/63) were from within Lusaka, and 13% (8/63) were from referring hospitals from other districts. However, for seven samples, one or all of the demographic information such as age, sex and residence was missing. Figure 1 depicts the administrative wards (highlighted in light blue) where the health facilities are located within Lusaka district which referred the patients for treatment to the University Teaching hospital.

### 2.2. Resistance Profiles

Among the 63 samples, 60 (95%) samples had mutations associated with MDR-TB, 2 (3%) rifampicin resistance only and 1 (2%) polyresistant as shown in Table 1. Among the MDR strains, 7% (4/60) had mutations associated with pre-XDR. None of the samples had mutations in the *rrs* and *gyrB* genes. Among the studied strains, 8% (5/63) had mutations in fluoroquinolone resistance related genes 3/5, gyrA Asp94Gly, 1/5, Asp94His, and 1/5, Gly88Cys (Table 1). Whilst four of the five strains that had mutations associated with fluoroquinolone resistance were phenotypically resistant to moxifloxacin and levofloxacin (Table 2). None of these four isolates phenotypically resistant to fluoroquinolones were resistant to bedaquiline and clofazimine (Table 2). Additionally, among the whole genome sequenced fifty strains, four and one strain had mutations associated with ethionamide (fabG-15C>T, ethA Thr61Met, 1215_1224del and 1412_1413insGG) and para-amino-salicylic acid (PAS) (folC Glu40Gly) resistances, respectively (Table 3 and Supplementary Table S1). Moreover, more drug-resistance-associated mutations were identified to drugs such as pyrazinamide (11/50), ethambutol (20/50) and streptomycin (15/50). One polyresistant strain had fluoroquinolone, isoniazid, pyrazinamide, and ethionamide resistant associated mutations but not MDR. The resistance profiles of the fifty whole genome sequenced strains are as shown in the Supplementary Table S1 and a summary in Table 3. Additionally, the distribution of the resistance patterns of these strains as per referring health facility are shown in Figure 1A.

### 2.3. Genotypes, Phylogeny and Clustering

**Figure 2:**
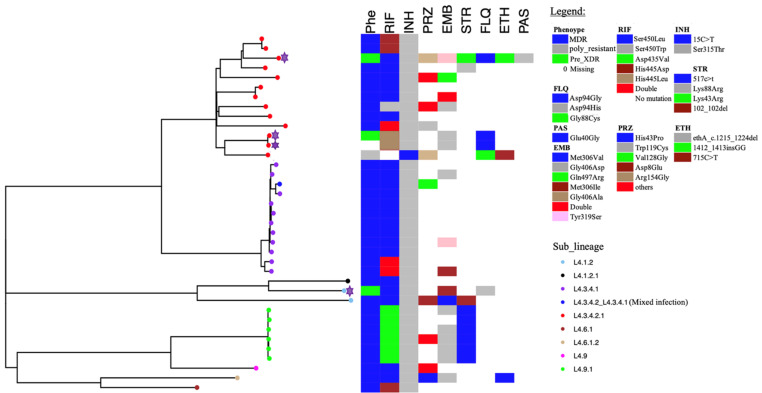
Maximum likelihood phylogenetic tree of studied strains. Phe represents phenotypic drug susceptibility patterns. For phenotypic drug susceptibility heatmap, blue represents multidrug resistance, green for pre-XDR, grey for poly-resistance while blank spaces indicate missing information. Whereas for genotypic susceptibility patterns, colored and blank boxes indicate the presence and absence of the drug-resistance-associated mutations, respectively. The pre-XDR strains are highlighted by the purple stars. The sub-lineages are indicated by the colored tips and labeled as shown in the legend. The letters represent the following: RIF = rifampicin, INH = isoniazid, EMB = ethambutol, STR = streptomycin, PRZ = pyrazinamide, FLQ = fluoroquinolone, ETH = ethionamide, PAS = para-amino salicylic acid.

**Figure 3:**
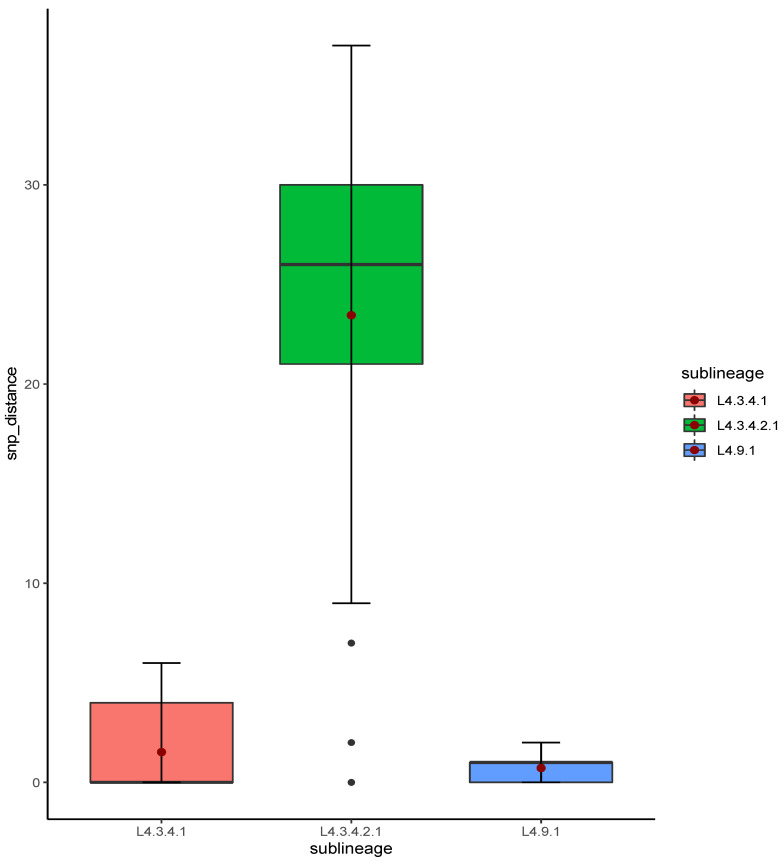
Box plot of SNP distances among strains from the studied major sub-lineages. The red dot represents the mean SNP difference for each sub-lineage.

The relatedness of the sequenced strains by WGS was illustrated by the maximum likelihood phylogenetic tree (Figure 2). All the 50 whole genome sequenced strains belonged to lineage 4 with the majority 38% (19/50) belonging to sub-lineage L4.3.4.2.1 followed by L4.3.4.1 which represented 32% (16/50). Supplementary Table S1 indicates the lineages and main spoligotypes of all the studied strains according to Coll et al., 2014 [21]. Of the four pre-XDR strains, three (3/4) belonged to L4.3.4.2.1 sub-lineage while the other one to L4.1.2 sub-lineage. Additionally, polyresistant strain also belonged to L4.3.4.2.1 sub-lineage. Phylogeny showed two out of the five strains with mutations in genes associated with fluoroquinolone resistance as clustered (with no SNP difference), whereas the other three were unique strains. Strains belonging to L4.3.4.2.1 sub-lineage were the most diverse with an average SNP difference of 23 SNPs, ranging from 1 to 37 SNPs when compared to other major sub-lineages in this study (Figure 3). Sub-lineage L4.9.1 had an average SNPs difference of one SNP with the range from zero to two SNPs, whereas L4.3.4.1 had an average of two SNPs difference with the range from zero to six SNPs. Overall, 64% (24/37) strains existed in clusters. Thirteen strains had poor coverage and hence they were excluded from clustering and phylogenetic analysis. The distribution of the sub-lineages according to the referring health facility in Lusaka, are as shown in Figure 1B.

#### Phylogenetic Analysis of Global Strains Belonging to Sub-Lineages L4.3.4.1 and L4.3.4.2.1

The downloaded sequences were confirmed by TB-profiler to belong to either sub-lineage L4.3.4.1 or sub-lineage L4.3.4.2.1. The phylogenetic trees showed that Zambian sub-lineage L4.3.4.1 strains made a cluster and were more closely related to United Kingdom, South African and Switzerland strains (Supplementary Figure S1A). While sub-lineage L4.3.4.2.1 strains were distributed throughout the tree (Supplementary Figure S2).

## 3. Discussion

In this study, we detected the emerging strains of pre-XDR TB in Lusaka, Zambia. Among the studied MDR strains, 4/60 (7%) were pre-XDR (Table 1). One strain with identity number E (Table 2) had mutations associated with pre-XDR although the phenotypic information was missing. Comparably, the percentage (7%) of pre-XDR strains in this study was slightly higher than that observed on the northern part of Zambia (1.7%) [22]. The difference may be due to a smaller sample size and selection criteria considered in our study. Other studies have reported the prevalence rate of pre-XDR among MDR-TB strains to be 27% and 16.7% in Zimbabwe [23] and Nigeria [24], respectively. The lower prevalence of pre-XDR in Zambia when compared to other countries could be that drug susceptibility testing and initiation of patients on second-line TB drugs, are only performed in very few designated health facilities in the country due to inadequate diagnostic capacity in other facilities. As a result, less patients are identified as some patients fail to visit the referral facilities due to resource constraints. Furthermore, our studied strains showed a mutation causing amino acid substitution Asp94Gly associated with fluoroquinolone resistance as being the most frequent (3/5). This suggested the significance of *gyrA* mutations in the development of fluoroquinolone resistance in *M. tuberculosis* as indicated by many studies [25,26,27]. Hence, the rapid detection of mutations in *gyrA* gene can lead to the early detection of fluoroquinolone-resistant strains which can have an influence on treatment regimens for patients.

All whole-genome-sequenced strains in this study belonged to lineage 4 with the predominant sub-lineage 4.3.4.2.1 (38%, 19/50). Furthermore, among the 4 pre-XDR strains, 3/4 belonged to sub-lineage 4.3.4.2.1, and 1 to sub-lineage 4.1.2. L4 and mainly sub-lineage 4.3.4.2.1 has been reported to be the most prevalent strains among the Zambian population [18,20]. Hence, a report of pre-XDR strains existence among the predominant strain is a source of concern as this is a potential threat to TB control.

Of the five strains with mutations in genes associated with fluoroquinolone resistance, two strains belonging to sub-lineage 4.3.4.2.1 were clustered suggesting recent transmission (Figure 2). Nevertheless, sub-lineage 4.3.4.2.1, which had most of the strains (4/5) with mutations associated with fluoroquinolone resistance, were less clustered (23 average SNP distances) when compared with strains belonging to sub-lineage 4.9.1 and sub-lineage 4.3.4.1. The average SNP distances for sub-lineages 4.9.1 and 4.3.4.1 were one and two SNP distances, respectively (Figure 3). This suggested that the spread of the majority of the sub-lineage 4.3.4.2.1 pre-XDR strains was mainly due to independent acquisition of drug-resistant-associated mutations [28]. This may be the result of poor-compliance to medications, or inadequate treatment given to the patients [7]. While the high overall clustering rate of 64% (24/37) is indicative of recent transmission among MDR-TB strains similar to the previous report in Lusaka [19]. Moreover, the three patients who were resistant to levofloxacin were still being treated with the regimen which had this drug (Table 2). This indicated the inadequacy of the treatment regimen which may lead to resistance amplification and consequently XDR-TB [29]. On the other hand, this demonstrated the need for improved testing capacity for optimization of treatment options.

Additionally, four and one strain had mutations associated with drug resistance to ethionamide (fabG1-15C>T, ethA Thr61Met, 1412_1413insGG and 1215_1224del) and para-amino salicylic acid (PAS) (folC Glu40Gly) (Table 3). PAS is rarely used in Zambia or in the neighboring countries for the treatment of drug-resistant tuberculosis and is considered as last-choice drug among the second-line group due to its very low effectiveness, adverse events and high price [10,30,31,32,33,34]. Therefore, a more detailed treatment history is needed for the patient from which the strain with a drug-resistant-associated mutation was taken for thorough investigations. Furthermore, this highlights the need to improve drug susceptibility testing to second-line drugs especially fluoroquinolones to prevent the spread and selection of drug-resistant strains. Fluoroquinolones are not only used for the treatment of drug-resistant tuberculosis in Zambia, but also for other bacterial infections. Thus, in some cases *M. tuberculosis* strains might be indirectly exposed to the fluroquinolones while the patient is being treated for other infections which may lead to resistance development. In our study, one strain although not MDR, was resistant to moxifloxacin and levofloxacin. The strain might have become resistant while the patient was being treated for TB with levofloxacin or due to the exposure of the strain to fluoroquinolones such as moxifloxacin used for the treatment of other unrelated conditions. It is worth noting that cross resistance occurs among the fluoroquinolones [35,36]. Additionally,, fluoroquinolones can be accessed over the counter by communities without prescriptions in Zambia leading to their misuse in some cases [37]. This inappropriate use could hasten the premature development of fluoroquinolone resistance in rifampicin-susceptible *M. tuberculosis* strains. Nevertheless, a majority of the studies indicate the fluoroquinolone resistance in rifampicin-susceptible TB as uncommon when compared to MDR TB [38,39,40]. Additionally, no resistance mutations associated with drugs such as kanamycin, amikacin, or capreomycin were detected in this study. One reason could be that these drugs being injected [41] are rarely abused by the communities and are not readily available in local pharmacies [42] when compare to the fluroquinolones.

As anticipated, WGS revealed more drug-resistance-associated mutations than targeted gene sequencing. This on the other hand emphasized the necessity of utilizing advanced methods like WGS for the thorough detection of drug-resistant mutations and to understand TBs genetic diversity. Additionally, the detection of pre-XDR strains and drug-resistance-associated mutations to ethionamide and PAS by WGS among a small number of screened samples, implied that possibly some cases with drug-resistance have gone unnoticed in health-care facilities. This in turn has implications for patient treatment and outcomes. Therefore, a large-scale study is required to provide more insight on the drug-resistant mutations especially to second line drugs and diversity of *M. tuberculosis* in an urban setup such as Lusaka, Zambia. This will facilitate the establishment of the role of specific genotypes in driving fluroquinolone resistance in Zambia and guide treatment options.

When compared with global strains, the Zambian strains belonging to sub-lineage L4.3.4.1, except for one, formed a mono-phylogenetic clade emphasizing the close relations between these strains. They also showed relations with the United Kingdom, South Africa and Switzerland strains. On the other hand, strains belonging to sub-lineage L4.3.4.2.1 were dispersed throughout the phylogenetic tree but were more closely related to Malawian and British strains, as was the case with sub-lineage 3.1.1, which was reported previously [43].

## 4. Materials and Methods

### 4.1. Sample Collection

Sixty-three (63) drug-resistant banked isolates collected from routine patients that visited the University Teaching Hospital (UTH) between January 2018 and December 2019 were used. Isolates were obtained from samples cultured using the MGIT^TM^ (Mycobacteria Growth Indicator Tube) system (Becton Dickson and Co., Franklin Lakes, NJ, USA) as per manufacturer’s instructions. Briefly, sputum samples were homogenized using a final concentration of 2% sodium hydroxide-N-acetyl- L-cysteine and 0.5 mL of the sediment was inoculated in MGIT^TM^ tubes containing 0.8 mL of PANTA growth supplement and incubated at 37 °C in BACTEC^TM^ 960 instruments. Broth from MGIT^TM^ tubes yielding *M. tuberculosis* isolates was aliquoted and stored in 20% glycerol at −80 °C.

**Figure 4:**
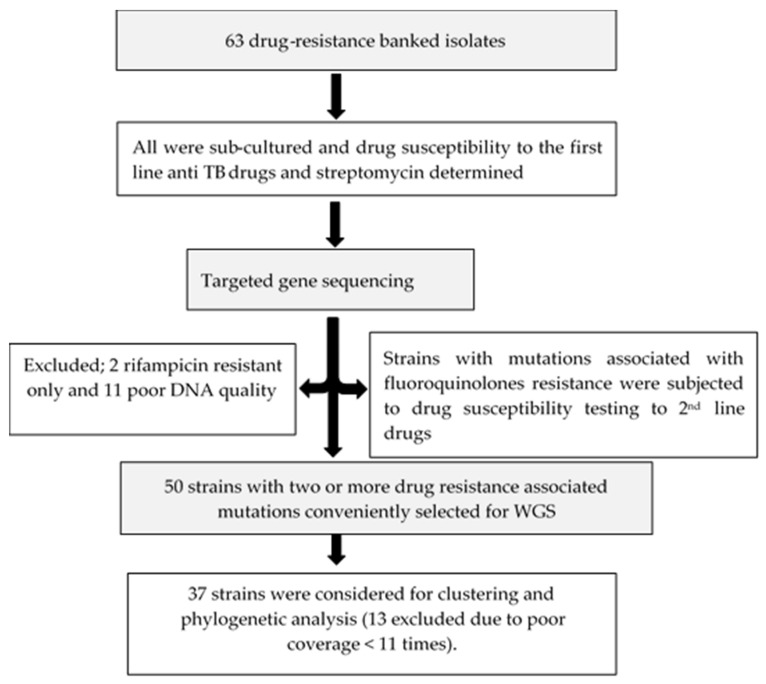
Flow chart summarizing the methods of this study.

The facility (UTH) is one of the three TB-specialized diagnostic centers in the country which also serves as a national reference hospital. Figure 4 shows the summarized workflow of the methods in this study.

### 4.2. Drug Susceptibility Testing and DNA Extraction

All sixty-three samples were sub-cultured in a Biosafety Level 3 (BSL3) laboratory, and drug susceptibility testing to first-line anti-tuberculosis and streptomycin was conducted to confirm their resistance patterns. Briefly, samples were removed from the freezer, thawed on ice and inoculated 2 to 3 drops in media and incubated at 37 °C until growth or no growth was detected in BACTEC™ 960 MGIT™ following the manufacturer’s instructions and as described previously [18]. The drug concentrations were as follows: rifampicin (RIF) 1.0 μg/mL, isoniazid (INH) 0.1 μg/mL, ethambutol (EMB) 5.0 μg/mL and streptomycin (STR) 1.0 μg/mL. DNA was extracted by heating method. Briefly, 1 mL of culture broth was aliquoted from an MGIT tube into cryovials heated at 90 °C for ten minutes in a dry heating block and then centrifugated at 15,000 revolutions per minute (rpm) at 4 °C for 5 min to extract supernatant for analysis. DNA was then shipped to the International Institute for Zoonosis Control in Japan for further analysis.

### 4.3. Targeted Sequencing of Drug-Resistance Associated Genes

Using specific primers (Table 4), polymerase chain reaction (PCR) was performed on all the samples for *rpoB, katG, inhA* promoter region, *rrs, gyrA*, and *gyrB* genes. Briefly, a 20 μL mixture of 5 M betaine, 25 mM deoxy-ribonucleotide triphosphate (dNTP), GoTaq buffer (Promega Corp, Madison, WI, USA), 1U of GoTaq DNA polymerase (Promega Corp), 10 μM of gene specific primers as described previously [44], and 1 μL of DNA template was used to conduct the PCR. The following conditions were used to carry out the reaction in a thermal cycler (Bio Rad Labs, Hercules, CA, USA): pre-heating at 96 °C for 60 s, 35 cycles of denaturation at 96 °C for 10 s, attachment at 55 °C for 10 s, elongation at 72 °C for 30 s and a post-run at 72 °C for 5 min. The amplicons were run on 2% agarose gel with 2 log-ladders at 80 volts for 25 min to confirm the targeted bands. PCR products were then purified using ExoSAP-IT^TM^ Express PCR Product Cleanup (Affymetrix Incorporated, Santa Clara, CA, USA) and Sanger sequencing was performed on an ABI 3500 Genetic Analyzer (Life Technologies Corp, Van Allen Way Carlsbad, CA, USA) as described previously [18]. The resulting sequences were mapped to the *M. tuberculosis* H37Rv reference sequence to manually identify the specific nucleotide differences in genes associated with drug resistance to anti-TB drugs. The following genes were analyzed; *rpoB* for rifampicin, *katG* and *inhA* promoter region for isoniazid, *rrs* for kanamycin, *gyrA* and *gyrB* genes for fluoroquinolones resistances. This was run using Geneious v 10.2.6, Biomatters, Ltd., Auckland, New Zealand https://www.geneious.com (accessed on 3 February 2023).

### 4.4. Whole Genome Sequencing

- Library preparation and sequencing

From the 63 samples, 50 with two or more mutations associated with drug resistance were conveniently selected for WGS. Two only resistant to rifampicin and eleven with poor DNA quality were excluded for further analysis. DNA quantity for each sample was determined using Qubit dsDNA HS (High Sensitivity) assay kits (Thermo Fisher Scientific, Waltham, MA, USA) following the manufacturer’s instructions.

Library preparations were carried out following Nextera XT library preparation kit (Illumina Inc., San Diego, CA, USA) and sequencing performed using illumina Miseq 2500 platform.

b. Data Analysis: Clustering, phylogenetic analysis and resistance patterns

The sequences generated via whole genome sequencing were analyzed for resistance patterns, clustering and phylogenetic analysis as previously described [43]. Briefly, the raw reads were quality controlled using FastQC v0.11.9 [45] before and after trimming for removal of adapter sequences and the low-quality reads using Trimmomatic v0.39 [46] (SLIDINGWINDOW:4:20 MINLEN:20). The results from FastQC were aggregated by multiQC [47]. The trimmed reads were mapped against the reference strain *M. tuberculosis* H37Rv (GenBank accession number: NC_000962.3) by Burrows Wheel Aligner (BWA), and manipulated with SAMtools through the snippy pipeline [48]. And as part of the snippy pipeline, variant calling was carried out with freebayes, and variant annotation performed with SnpEff. Full and core genome alignments were then generated with Snippy core. The full genome alignment was uploaded to Genealogies Unbiased By recomBinations In Nucleotide Sequences (Gubbins) [49] to generate an alignment with filtered polymorphic sites.

A maximum likelihood phylogenetic tree was then constructed using RAxML with 1000 bootstrap and GTR+F model of evolution in IQ-tree [50]. The ModelFinder in IQ-tree was used to identify the appropriate model of evolution [51]. The tree was visualized by R package ggtree [52,53]. The sequences considered for the phylogenetic tree had an average coverage of 33 and ranged from 11 to 101 times. Thus, 13 strains were excluded. Cluster analysis was executed on a multisequence alignment containing variant core-genome sites for the three major sub-lineages in this study. For statistical analysis, the multisequence alignment was evaluated for SNP distances in R-studio using the APE package [54,55]. Boxplots were utilized to illustrate SNP distances for the three major sub-lineages. A genomic cluster was defined as strains with less or equal to 12 SNPs differences between them [56,57]. While clustering rate was calculated as the number of clustered strains divided by the total number of the analyzed strains [19].

TB-profiler v. 3.0.3 [17] and Galaxy platform [58] were used to classify the strains to the lineage and sub-lineages level including the identification of drug-resistant-associated mutations.

c. Phylogenetic analysis of global strains belonging to sub-lineages L4.3.4.1 and L4.3.4.2.1

A total of 1213 global strains were downloaded from European Nucleotide Archive (ENA) browser using most of the accession numbers from the TB-profiler. Of these 1198 were successfully processed, 354 belonged to sub-lineage L4.3.4.1 and 844 strains L4.3.4.2.1. Briefly the downloaded reads were checked for quality with fastQC and trimmed for low-quality reads using Trimmomatic v0.39. The trimmed reads were further processed using snippy pipeline to generate the alignments. *M. tuberculosis* BED mask file from Snippy-package was used to filter SNPs in PE/PPE gene families to prevent false hits in repetitive regions. Gubbins was used to generate the phylogenetic trees under the default settings. The sub-lineages were determined and confirmed using TB-profiler v. 3.0.3 [17]. The trees were then visualized in R-studio using R package ggtree [52,53].

### 4.5. Drug Susceptibility Testing to Second Line Drugs

Four strains with mutations in the fluoroquinolone resistance associated genes detected through targeted sequencing were subjected to phenotypic drug susceptibility testing to second line drugs using BACTEC™ 960 MGIT™ system (Becton Dickson and Co) at concentrations; bedaquiline (1.0 μg/mL), levofloxacillin (0.25 μg/mL), moxifloxacillin (0.25 μg/mL) and clofazimine (1.0 μg/mL).

## 5. Conclusions

In view of the emerging pre-XDR strains demonstrated in our study and the fact that cross-resistance exists among fluoroquinolones [35,36], we recommend, where possible, for TB control programs to emphasize the routine screening of drug-resistant patients for second-line drugs susceptibility patterns before or immediately after initiating treatment as this will stop the progression of pre-XDR to XDR-TB and improve patient treatment outcome.

## Figures and Tables

**Table 1:** Percentages of drug-resistance-associated mutations based on targeted gene sequencing for the sixty-three samples. The percentages have been rounded-off to whole numbers.

		Mutations	Number	Percentage (%)
Rifampicin		Asp435Val ^a^	8	13
		His445Asp ^a^	7	11
		Ser450Leu ^a^	34	54
		Rifampicin mutation only (His445Tyr ^a^)	2	3
		No mutation	1	2
		Other rifampicin resistance mutations	11	17
Isoniazid		Ser315Thr ^b^	60	95
		15 C>T ^c^	1	2
		No mutation	2	3
MDR		Asp435Tyr ^a^, Ser315Thr ^b^	2	3
		Asp435Val ^a^, Ser315Thr ^b^	8	13
		His445Asp ^a^, Ser315Thr ^b^	7	11
		Ser450Leu ^a^, Ser315Thr ^b^	30	48
		Other MDR mutation combinations	9	14
		Others (mono and polyresistant)	3	5
		Ser450Leu ^a^, Ser315Thr ^b^, Asp94His ^d^	1	2
	Pre-XDR	Ser450Leu ^a^, Ser315Thr ^b^, Asp94Gly ^d^	1	2
		His445Leu ^a^, Ser315Thr ^b^, Asp94Gly ^d^	2	3
Polyresistant		15 C>T ^c^, Gly88Cys ^d^	1	2
		Non polyresistant	62	98

^a^: rpoB, ^b^: katG, ^c^: inhA regulatory region, ^d^: gyrA.

**Table 2:** Pre-XDR and other strains that had mutations in genes associated with drug resistance to second-line drugs.

			Drug Susceptibility Pattern									Gene Mutations										Treatment				
ID	Age	Sex	I	R	E	S	Bdq	Lfx	Mfx	Cfz	Phenotype	*gryA*	*rpoB*	*katG*	*inh_promoter*	*pncA*	*embB*	*rpsL*	*ethA*	*folC*	Sublineage					
A	41	M	X	√	√	√	√	X	X	√	poly-resistant	G88C	-	-	15C>T	R154G	-	-	15C > T	-	L4.3.4.2.1	Lfx	I	R	E	Z
B	29	M	X	X	√	√	√	X	X	√	pre-XDR	D94H	S450L	S315T	-	-	M306I	-	-	-	L4.1.2	Lfx	Bdq	Lzd	Cs	-
C	48	F	X	X	√	√	√	X	X	√	pre-XDR	D94G	S450L	S315T	-	R154G	Y319S	K43R	1412_1413insGG	E40G	L4.3.4.2.1	-	-	-	-	-
D	24	F	X	X	√	√	√	X	X	√	pre-XDR	D94G	H445L	S315T	-	-	M306V	-	-	-	L4.3.4.2.1	Lfx	Km	Eto	Cs	-
E	-	-	-	-	-	-	-	-	-	-	* pre-XDR	D94G	H445L	S315T	-	-	M306V	-	-	-	L4.3.4.2.1	-	-	-	-	-
F	42	M	X	X	X	X	-	-	-	-	MDR	-	S450L	S315T	-	H43P	M306V	-	1215_1224del	-	L4.6.1.2	Lfx	Km	Eto	Cs	Z
G	27	F	X	X	√	√	-	-	-	-	MDR	-	S450L	S315T	-	-	-	-	T61M	-	L4.3.4.2.1	-	-	-	-	-

I—isoniazid, R—rifampicin, E—ethambutol, Z—pyrazinamide, S—streptomycin, Bdq—bedaquiline, Lfx—levofloxacin, Mfx—moxifloxacin, Cfz—clofazimine, Lzd—Linezolid, Cs—cycloserine, Km—kanamycin, Eto—ethionamide, ID for identity number,—no mutation or missing information, X resistant drug susceptibility pattern, √ susceptible drug susceptibility pattern, * pre-XDR- strain defined as pre-XDR based on the presence of mutations in the resistance associated genes as phenotypic drug, susceptibility patterns were not conducted.

**Table 3:** Summary of whole genome sequencing results for fifty samples for drug-associated mutations not covered by targeted sequencing.

	Drug	Mutations	Number	Percentage (%)
Non-MDR	Polyresistant	Gly88Cys, 15 C>T, Arg154Gly	1	2
	Pyrazinamide (Z)	Arg154Gly	1	2
		Trp119Cys	1	2
		His43Pro	1	2
		Val128Gly	1	2
MDR plus		Other pyrazinamide mutations	6	12
		No mutations	40	80
	Ethambutol (E)	Met306Val	2	4
		Met306Val	4	8
		Met306Ile	3	6
		Tyr319Ser	3	6
		Other ethambutol mutations	8	16
		No mutations	30	60
	Streptomycin (S)	Lys88Arg	6	12
		Other streptomycin mutations	9	18
		No mutations	35	70
	Ethionamide (Eto)	Thr61Met	1	2
		Ser450Leu, Ser315Thr, 15C>T	1	2
		Other ethionamide mutations	2	4
		No mutations	46	92
	Para-amino salicylic acid (PAS)	Glu40Gly	1	2
		No mutations	49	98

**Table 4:** Nucleotide sequences of primers used in this study for targeted sequencing.

*Gene*	Primer Set	
*rpoB*	Foward	CAGGACGTGGAGGCGATCAC
	Reverse	GAGCCGATCAGACCGATGTTGG
*katG*	Foward	ATGGCCATGAACGACGTCGAAAC
	Reverse	CGCAGCGAGAGGTCAGTGGCCAG
*inhA*	Foward	TCACACCGACAAACGTCACGAGC
	Reverse	AGCCAGCCGCTGTGCGATCGCCA
*gyrA*	Foward	AGCGCAGCTACATCGACTATGCG
	Reverse	CTTCGGTGTACCTCATCGCCGCC
*gryB*	Foward	CGGCACGTAAGGCACGAGAG
	Reverse	GAACCGGAACAACAACGTCAAC
*rrs*	Foward	CGGATCGGGGTCTGCAACTCGAC
	Reverse	CAAGAACCCCTCACGGCCTACG

## Data Availability

Data used in this study can be accessed by request from the corresponding author. While the publicly available data utilized, can be found on https://tbdr.lshtm.ac.uk/sra. accessed on 13 May 2022.

## Supplementary Materials

The following are available online at https://www.mdpi.com/article/10.3390/antibiotics12071126/s1, Figure S1: Maximum likelihood tree of the global L4.3.4.1 sub-lineage. The nodes are highlighted according to the country of origin of the sequences. The nodes for the Zambian sub-lineage L4.3.4.1 strains are in red color forming the monophyletic clade; Figure S2: Phylogenetic tree of the Zambian strains with global sub-lineage L4.3.4.2.1 strains. The nodes for Zambia sub-lineage L4.3.4.2.1 strains are in red color dispersed throughout the phylogenetic tree; Table S1: Figure showing the fifty (50) studied samples with their resistance patterns, lineage, main spoligotypes, total sequences, mean coverages, number of reads and the inclusion in the tree analysis. The ** Lineage and ** main spoligotype was as described by Coll et al., 2014 [21].
