# Supplementary material for: Genomic Analysis of Mycobacterium tuberculosis Strains Resistant to Second-Line Anti-Tuberculosis Drugs in Lusaka, Zambia
Source: Antibiotics (Basel). 2023 Jun 29;12(7):1126. doi: 10.3390/antibiotics12071126 (PMC10376136; doi:10.3390/antibiotics12071126)
Supplement: Supplementary file 1 [file antibiotics-12-01126-s001.zip › Figure.pdf]

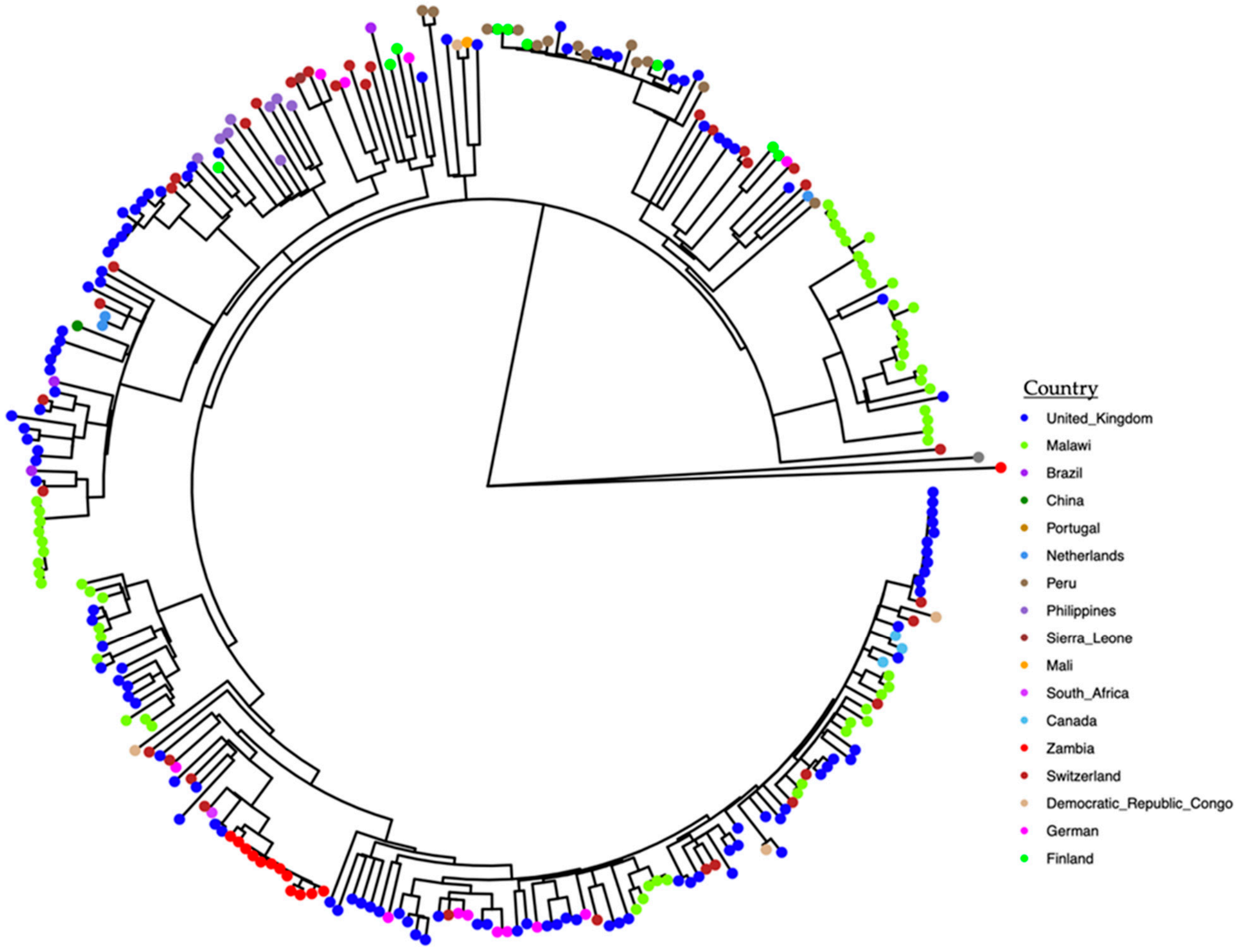

Supplementary Figure S1: Maximum likelihood tree of the global L4.3.4.1 sub-lineage. The nodes are highlighted according to the country of origin of the sequences. The nodes for the Zambian sub-lineage L4.3.4.1 strains are in red color forming the monophyletic clade.

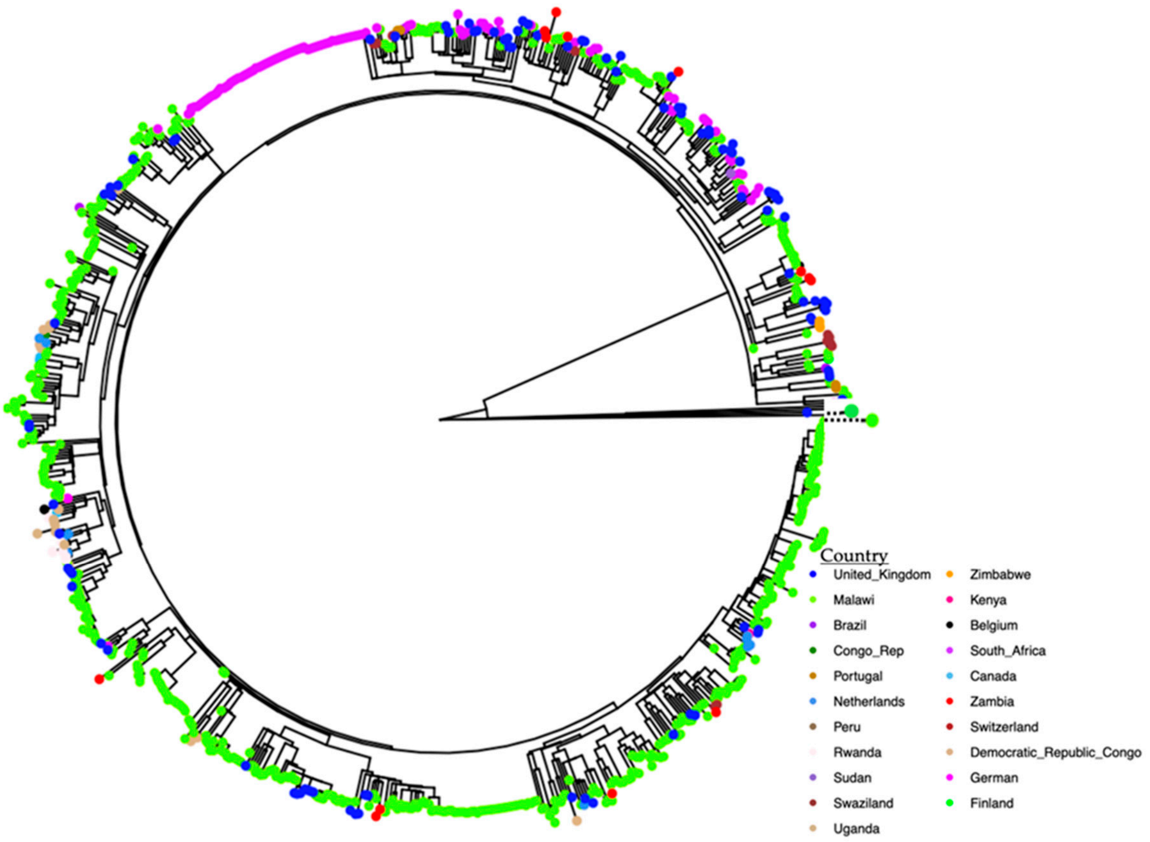

Supplementary Figure S2: Phylogenetic tree of the Zambian strains with global sub-lineage L4.3.4.2.1 strains. The nodes for Zambia sub-lineage L4.3.4.2.1 strains are in red color dispersed throughout the phylogenetic tree.
